# Phase 1 clinical trial of the PI3Kδ inhibitor YY-20394 in patients with B-cell hematological malignancies

**DOI:** 10.1186/s13045-021-01140-z

**Published:** 2021-08-23

**Authors:** Bo Jiang, Junyuan Qi, Yuqin Song, Zengjun Li, Meifeng Tu, Lingyan Ping, Zongliang Liu, Hanying Bao, Zusheng Xu, Lugui Qiu

**Affiliations:** 1 State Key Laboratory of Experimental Hematology, National Clinical Research Center for Hematological Disorders, Institute of Hematology and Blood Diseases Hospital, Chinese Academy of Medical Sciences and Peking Union Medical College, No. 288 Nanjing Road, Heping District, Tianjin, 3000241 China; 2 Key Laboratory of Carcinogenesis and Translational Research, Department of Lymphoma, Peking University School of Oncology, Beijing Cancer Hospital and Institute, Beijing, China; 3 Shanghai Yingli Pharmaceutical Co., Ltd, Shanghai, China

**Keywords:** Linperlisib, PI3Kδ inhibitor, Dose-limiting toxicity, Non-Hodgkin’s lymphoma, Pharmacokinetics

## Abstract

YY-20394, an oral phosphatidylinositol 3-kinase delta (PI3Kδ) inhibitor, was investigated in a first-in-human study of patients with relapsed or refractory B-cell malignancies. During dose escalation, 25 patients received 20–200 mg of YY-20394 daily. The primary outcome measures were tolerability and dose-limiting toxicity (DLT). The secondary outcomes were pharmacokinetic parameters, progression-free survival (PFS) and the objective response rate (ORR). Since no patients experienced DLT, the maximum tolerated dose (MTD) was not reached. The majority (≥ 5%) of drug-related adverse events were ≥ grade III, being neutropenia (44.0%), pneumonia (16.0%), hyperuricemia (12.0%), lymphocythemia (8.0%), leukopenia (8.0%) and pneumonitis (8.0%). The overall ORR was 64.0% (95% confidence interval (CI): 45.2, 82.8%) including 5 patients with complete remission (CR), 11 with partial remission (PR), 2 with stable disease (SD) and 7 with progressive disease (PD), while the disease control rate (DCR) was 72.0% (95% CI: 54.4, 89.6%). The ORR of 10 patients with follicular lymphoma was 90%. The median PFS time was 255 days. One PR patient with chronic lymphocytic leukemia/small lymphocytic lymphoma (CLL/SLL) who received 40 mg q.d. had a durable response of around 36 months. The median PFS time of 10 patients with follicular lymphoma was 300 days. A recommended phase 2 dose of 80 mg q.d. was established. Considering that YY-20394 was well-tolerated with promising preliminary efficacy, further development is warranted.

*Trial registration* clinicaltrials.gov, NCT03757000, retrospectively registered, November 28, 2018, https://clinicaltrials.gov/ct2/show/NCT03757000?term=NCT03757000&draw=2&rank=1.

**Supplementary Information:**

The online version contains supplementary material available at 10.1186/s13045-021-01140-z.

To the editor

The selective PIP2 3-kinase δ (PI3Kδ) inhibitor idelalisib in combination with rituximab [1–3] and the PI3K-δ/γ inhibitor duvelisib (IPI-145) [4] have been approved to treat B-cell malignancies. In addition, a selective PI3Kδ inhibitor parsaclisib (INCB050465) is undergoing phase 2 trials [5, 6]. We report the first-in-human clinical investigation of YY-20394, a novel PI3Kδ-selective inhibitor, in a dose escalation study in patients with relapsed or refractory B-cell malignancies to evaluate its safety, pharmacokinetic (PK) parameters and efficacy.

YY-20394 [N-[5-[6-fluoro-8-[[4-(1-hydroxy-1-methylethyl)-1-piperidinyl]methyl]-2-(4-morpholinyl)-4-quinazolinyl]-2-methoxy-3-pyridinyl]-methanesulfonamide] is structurally different from idelalisib, and is a potent PI3Kδ inhibitor (IC_50_: 4.6 nM) with less activity against PI3Kγ giving a kinase inhibition profile that is more PI3Kδ-selective by nearly 2 orders of magnitude (Additional file 1: Table S1).

Patients ≥ 18 years old with refractory or relapsed B-cell malignancies were enrolled from November, 2017 to completion of the trial in November, 2019. Inclusion and exclusion criteria are listed in Additional file 2 and baseline patient characteristics in Additional file 3: Table S2.

Of the 27 enrolled patients, 25 were evaluable including 10 follicular lymphoma (FL), 4 mantle cell lymphomas (MCL), 4 chronic lymphocytic leukemia/small lymphocytic lymphoma (CLL/SLL), 2 diffuse large B-cell lymphoma (DLBCL), 3 DLBCL/FL, 1 marginal zone lymphoma (MZL) and 1 lymphatic plasma cell lymphoma (LPL) patients. During dose escalation, patients received YY-20394 tablets q.d. at dosages of 20, 40, 80, 140 or 200 mg. The maximum tolerated dose (MTD), dose escalation phase and dose-limiting toxicity (DLT) as well as hematological toxicity classifications are described in Additional file 4. The primary endpoints were safety, tolerability and the MTD of YY-20394. Secondary endpoints were PK parameters and efficacy. Response criteria followed the revised International Research Working Group (IRWG) for non-Hodgkin lymphomas (NHL) [7], and the International Working Group on Chronic Lymphocytic Leukemia (IWCLL) criteria for CLL [8]. Efficacy determinations were the objective response rate (ORR), disease control rate (DCR), complete remission (CR), partial remission (PR), stable disease (SD), progressive disease (PD) and progression-free survival (PFS).

The safety evaluation of YY-20394 included adverse events (AEs) and serious AEs (SAEs) by standard categorizations. All 25 patients had at ≥ 1 AE. Thirteen (52.0%) and 9 (36.0%) patients experienced SAEs and drug-related AEs. The drug-related AEs that occurred in ≥ 20% of patients were neutropenia (68.0%), leukopenia (44.0%), elevated lactate dehydrogenase (44.0%), elevated α-hydroxybutyrate dehydrogenase (24.0%), thrombocytopenia (20.0%) and hyperuricemia (20.0%). Those that occurred in ≥ 5% of patients are also listed (Table 1). Among 32 ≥ grade III AEs, most were grade III; 3 cases of grade IV hyperuricemia and 4 grade IV neutropenia, but no grade V AEs occurred. Overall, YY-20394 had a manageable safety profile. It is noteworthy that unlike other PI3K inhibitors, the incidence of diarrhea, colitis, and hepatotoxicities [9] was very low.

**Table 1:** Drug-related adverse events occurring in ≥ 5% of evaluable patients at grade III or greater

Drug-related adverse events categorized by SOC and PT	Number of patients with grade I/II at > 5% incidence	Number of patients with ≥ grade III
*Hematological*		
Neutropenia	17 (68.0)	11 (44.0)
Leukopenia	11 (44.0)	2 (8.0)
Thrombocytopenia	5 (20.0)	1 (4.0)
Lymphocythemia	3 (12.0)	2 (8.0)
Anemia	3 (12.0)	0
Leukocytosis	2 (8.0)	0
*Non-hematological*		
Elevated serum lactate dehydrogenase	11 (44.0)	1 (4.0)
Elevated serum α-hydroxybutyrate dehydrogenase	6 (24.0)	1 (4.0)
Hyperuricemia	5 (20.0)	3 (12.0)
Upper respiratory tract infection	4 (16.0)	1 (4.0)
Pneumonia	4 (16.0)	4 (16.0)
Proteinuria	4 (16.0)	0
Hyperbilirubinemia	3 (12.0)	0
Elevated alanine aminotransferase	3 (12.0)	0
Elevated aspartate aminotransferase	2 (8.0)	0
Elevated serum alkaline phosphatase	3 (12.0)	0
Weight loss	3 (12.0)	0
Pneumonitis	3 (12.0)	2 (8.0)
Weight gain	2 (8.0)	1 (4.0)
Elevated γ-glutamyltransferase	2 (8.0)	0
Elevated bilirubin	2 (8.0)	0
Diarrhea	2 (8.0)	0
Cough	2 (8.0)	0
Oropharyngeal pain	2 (8.0)	0
Maculopapule	2 (8.0)	0
Fever	2 (8.0)	0
Fatigue	2 (8.0)	0

*AE* adverse event, *SOC* system organ class, *PT* preferred term

After single administrations of YY-20394 (20 to 140 mg), terminal elimination was consistent and in vivo exposure increased proportionally in a dose-dependent manner (C_max_, AUC_0-t_, AUC_0-∞_) (Additional file 5: Table S3). Also, the PK parameters after multiple administrations revealed that the exposure of YY-20394 (C_max_, AUC_0-t_, AUC_0-∞_) increased with dosage (20 to 200 mg) (Additional file 6: Table S4). The 80 mg dose level produced a serum concentration of YY-20394, corresponding to 90% inhibition of basophil activation in vitro.

YY-20394 treatment produced an overall 64.0% ORR (16/25) (95%confidence interval (CI): 45.2, 82.8%) and a 72.0% DCR (18/25) (95%CI: 54.4, 89.6%) in B-cell malignancies, including 5 CR, 11 PR, 2 SD and 7 PD cases. Notably, in the FL patients, a 90% ORR (9/10) (95%CI: 71.4, 100.0) and 90% DCR (9/10) (95%CI: 71.4, 100.0) were found (Fig. 1a), with 3 CR (80 mg), 6 PR (1/40 mg and 5/80 mg) and 1 PD (200 mg) (Fig. 1b), with a median PFS time of 300 days. The median PFS was 255 days when all evaluable patients data were combined, with the longest treatment duration being 36 months (40 mg, CLL/ SLL patient) (Fig. 1c).

**Fig. 1:**
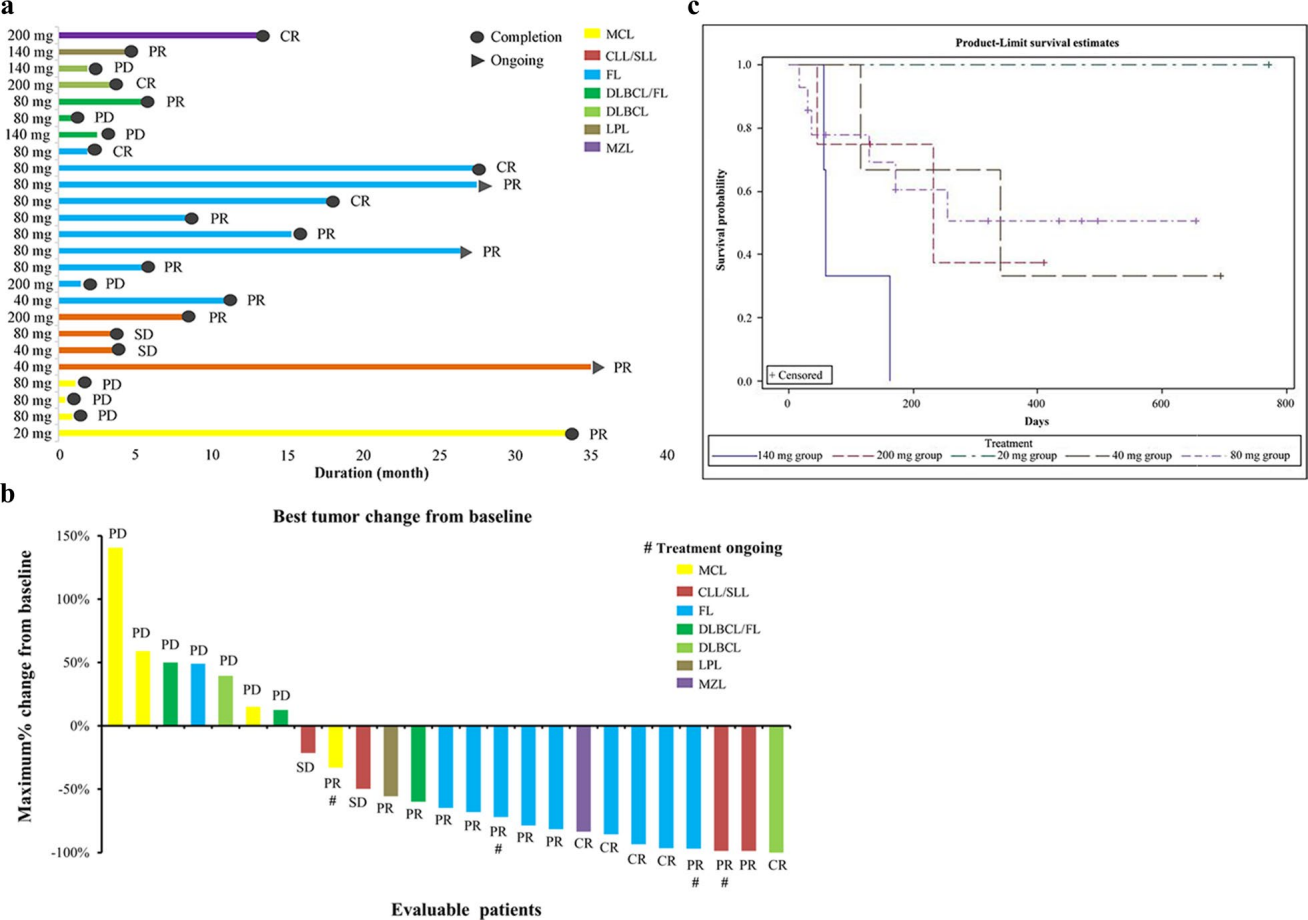
Efficacy evaluation of YY-20394 treatments in the dose escalation study of B-cell malignancies. **a** Overall efficacy chart of YY-20394. **b** Waterfall plot of overall tumor changes from baseline ^#^indicates transient staging with ongoing treatment at the end of the study period. **c** PFS curve (days) in the 5 patient dosing groups. *Note*: CLL/SLL, chronic lymphocytic leukemia/small lymphocytic lymphoma; CR, complete remission; DLBCL, diffuse large B-cell lymphoma; FL, follicular lymphoma; LPL, Lymphatic plasma cell lymphoma; MCL, mantle cell lymphoma; MZL marginal zone lymphoma; PD, progressive disease; PR, partial remission; SD, stable disease

From the combination of safety, PK, ORR and DOR data, the recommended phase 2 dose for YY-20394 monotherapy was established at 80 mg q.d.

With its excellent efficacy and tolerability in aggressive lymphomas the clinical development of YY-20394 as a novel treatment for relapsed or refractory hematological malignancies is warranted.

## Supplementary information

**Additional file 1.Table S1**: YY-20394 is highly selective in targeting PI3Kδ.

**Additional file 2.** Inclusion and exclusion criteria.

**Additional file 3.Table S2**: Basic characteristics of patients in each group.

**Additional file 4.** The methods and definition of MTD, dose escalation phase and DLT as well as hematological toxicity.

**Additional file 5.Table S3**: Mean pharmacokinetic parameters of patients after a single dose in each dosage group.

**Additional file 6.Table S4**: Mean pharmacokinetic parameters of patients after multiple administrations in each dosage group.

## Data Availability

The datasets used and/or analysed during the current study are available from the corresponding author on reasonable request.

## Abbreviations

AEs: Adverse events
CI: Confidence interval
CLL: Chronic lymphocytic leukemia
CR: Complete remission
DCR: Disease control rate
DLBCL: Diffuse large B-cell lymphomas
DLT: Dose-limiting toxicity
FL: Follicular lymphoma
IRWG: International Research Working Group
IWCLL: International Working Group on Chronic Lymphocytic Leukemia
MTD: Maximum tolerated dose
NHL: Non-Hodgkin lymphomas
ORR: Objective response rate
PD: Progressive disease
PFS: Progression free survival
PI3Kδ: Phosphatidylinositol 3-kinase delta
PK: Pharmacokinetic
PR: Partial remission
SAEs: Serious AEs
SD: Stable disease
SLL: Small lymphocytic lymphoma
