# Supplementary material for: Phase 1 clinical trial of the PI3Kδ inhibitor YY-20394 in patients with B-cell hematological malignancies
Source: J Hematol Oncol. 2021 Aug 23;14:130. doi: 10.1186/s13045-021-01140-z (PMC8381505; doi:10.1186/s13045-021-01140-z)
Supplement: Supplementary file 1 — Additional file 1.Table S1: YY-20394 is highly selective in targeting PI3Kδ. [file 13045_2021_1140_MOESM1_ESM.docx]

**Additional File 1: Table S1. YY-20394 is highly selective in targeting PI3Kδ**

| **Compounds** | **PI3Kδ IC_50_ (nM)** | **Ratio of IC_50_** | | |
| --- | --- | --- | --- | --- |
|  |  | **α/δ** | **β/δ** | **γ/δ** |
| YY-20394 | 4.6 | 252 | 30 | 1125 |
| Idelalisib | 4.5 | 227 | 40 | 13 |
