# Supplementary material for: Phase 1 clinical trial of the PI3Kδ inhibitor YY-20394 in patients with B-cell hematological malignancies
Source: J Hematol Oncol. 2021 Aug 23;14:130. doi: 10.1186/s13045-021-01140-z (PMC8381505; doi:10.1186/s13045-021-01140-z)
Supplement: Supplementary file 2 — Additional file 2. Inclusion and exclusion criteria. [file 13045_2021_1140_MOESM2_ESM.docx]

**Additional File 2. Inclusion and exclusion criteria**

The patients enrolled in the trial were ≥ 18 years old and diagnosed with refractory or relapsed B-cell malignancy, confirmed by histology and cytology investigations. The patients had: an Eastern Cooperative Oncology Group (ECOG) status between 0 and 2; a life expectancy of ≥ 3 months; ≥ 1 measurable lesion (only for the expansion period); good organ functions; all nucleate cell (ANC) ≥ 1.0 × 10^9^/L; platelet (PLT) ≥ 70 × 10^9^/L; hemoglobin (Hb) ≥ 80 g/L; left ventricular ejection fraction (LVEF) ≥ 50%; total bilirubin (TBIL) ≤ 1.5 × ULN; blood urea nitrogen (BUN) and creatinine (Cr) ≤ 1 × ULN; alanine transaminase (ALT) and aspartate transaminase (AST) ≤ 1.5 × ULN; and a Fridericia-corrected QT interval < 470 ms (females) and < 450 ms (males). Patients must have had a washout period of ≥ 4 weeks from the end of any previous antitumor treatment including hormone therapy or molecular targeted chemotherapy and also radiotherapy or surgery prior before receiving the first dose in the present trial.

Patients were excluded from the trial if they had previous progression during PI3Kδ targeted therapy (except discontinuation for intolerability); use of any other antitumor therapies within 4 weeks; third space effusion (i.e., an excessive amount of hydrothorax and/or ascites) that could not be controlled with drainage and/or other methods; use of steroids (prednisone equivalent) > 20 mg/day for more than 14 consecutive days; the patient could not swallow; had chronic diarrhea and/or intestinal obstruction; existing multiple conditions that could have affected drug intake and disposition; unable to suspend medications that may prolong QT interval during the study (e.g. antiarrhythmic agents); lymphoma with central nervous system invasion; allergic constitution, or known anaphylaxis to any component of this product; active viral, bacterial or fungal infection requiring treatment (e.g., pneumonia); known hepatitis B virus (HBV), hepatitis C virus (HCV) infection (HBV infection is defined as hepatitis B surface antigen (HBSAg) positive or HBSAg negative but HBV antibody positive with HBV DNA replication positive); medical history of acquired or congenital immunodeficiency diseases, organ or allogeneic bone marrow transplantation; had received autologous hematopoietic stem cell transplantation within 90 days before commencement of the trial; cardiac disease including (1) angina pectoris; (2) arrhythmias requiring drug therapy or were clinically significant; (3) myocardial infarction; (4) heart failure; (5) cardiac diseases deemed by investigators as rendering the patient unsuitable to participate in the trial; pregnant or lactating women or baseline pregnancy testing positive for fertile women; concomitant diseases (such as serious hypertension, diabetes mellitus, thyroid disease) seriously hazardous to the patient’s safety or completion of the study as judged by the investigator; received granulocyte colony stimulating factor (GCSF) or blood transfusion within 7 days prior to hematological examination in the screening period; or had another primary malignancy during the last 5 years.
