# Supplementary material for: Phase 1 clinical trial of the PI3Kδ inhibitor YY-20394 in patients with B-cell hematological malignancies
Source: J Hematol Oncol. 2021 Aug 23;14:130. doi: 10.1186/s13045-021-01140-z (PMC8381505; doi:10.1186/s13045-021-01140-z)
Supplement: Supplementary file 3 — Additional file 3.Table S2: Basic characteristics of patients in each group. [file 13045_2021_1140_MOESM3_ESM.docx]

**Additional File 3: Table S2. Basic characteristics of patients in each group**

| **Index** | **20 mg**  **(n = 1)** | **40 mg**  **(n = 3)** | **80 mg**  **(n = 14)** | **140 mg**  **(n = 3)** | **200 mg**  **(n = 4)** | **Total**  **(n = 25)** |
| --- | --- | --- | --- | --- | --- | --- |
| **Gender, n (%)** | | | | | | |
| Male | 1 (100.0) | 3 (100.0) | 9 (64.3) | 1 (33.3) | 2 (50.0) | 16 (64.0) |
| Female | 0 (0.0) | 0 (0.0) | 5 (35.7) | 2 (66.7) | 2 (50.0) | 9 (36.0) |
| **Nation, n (%)** |  |  |  |  |  |  |
| Han | 1 (100.0) | 3 (100.0) | 12 (85.7) | 2 (66.7) | 4 (100.0) | 22 (88.0) |
| Others | 0 (0.0) | 0 (0.0) | 2 (14.3) | 1 (33.3) | 0 (0.0) | 3 (12.0) |
| **Height (cm)** |  |  |  |  |  |  |
| N (Missing) | 1 (0) | 3 (0) | 14 (0) | 3 (0) | 4 (0) | 25 (0) |
| Mean ± SD | 167.0 ± | 170.0 ± 12.5 | 166.4 ± 8.6 | 165.0 ± 5.0 | 164.3 ± 14.4 | 166.3 ± 9.1 |
| Median | 167.0 | 174.0 | 168.5 | 165.0 | 166.0 | 167.0 |
| Q1, Q3 | 167.0, 167.0 | 156.0, 180.0 | 160.0, 173.0 | 160.0, 170.0 | 152.5, 176.0 | 160.0, 174.0 |
| Min, Max | 167.0, 167.0 | 156.0, 180.0 | 149.0, 176.0 | 160.0, 170.0 | 147.0, 178.0 | 147.0, 180.0 |
| **Weight (kg)** |  |  |  |  |  |  |
| N (Missing) | 1 (0) | 3 (0) | 14 (0) | 3 (0) | 4 (0) | 25 (0) |
| Mean ± SD | 57.5 ± | 65.7 ± 14.8 | 71.8 ± 14.6 | 73.1 ± 2.6 | 67.5 ± 14.4 | 70.0 ± 13.1 |
| Median | 57.5 | 69.7 | 67.6 | 72.0 | 62.1 | 68.0 |
| Q1, Q3 | 57.5, 57.5 | 49.3, 78.1 | 61.6, 83.0 | 71.2, 76.0 | 59.2,75.7 | 61.4, 76.0 |
| Min, Max | 57.5, 57.5 | 49.3, 78.1 | 51.6, 100.5 | 71.2, 76.0 | 57.0, 88.7 | 49.3, 100.5 |
| **ECOG score, n (%)** |  |  |  |  |  |  |
| 0 | 0 (0.0) | 0 (0.0) | 7 (50.0) | 1 (33.3) | 1 (25.0) | 9 (36.0) |
| 1 | 1 (100.0) | 3 (100.0) | 7 (50.0) | 2 (66.7) | 3 (75.0) | 16 (64.0) |
| **Lymphoma type, n (%)** |  |  |  |  |  |  |
| Follicular lymphoma | 0 (0.0) | 1 (33.3) | 8 (57.1) | 0 (0.0) | 1 (25.0) | 10 (40.0) |
| Chronic lymphocytic leukemia | 0 (0.0) | 2 (66.7) | 1 (7.1) | 0 (0.0) | 1 (25.0) | 4 (16.0) |
| Mantle cell lymphoma | 1 (100.0) | 0 (0.0) | 3 (21.4) | 0 (0.0) | 0 (0.0) | 4 (16.0) |
| Diffuse large B- cell lymphoma | 0 (0.0) | 0 (0.0) | 0 (0.0) | 1 (33.3) | 1 (25.0) | 2 (8.0) |
| Follicular lymphoma with diffuse large B-cell lymphoma | 0 (0.0) | 0 (0.0) | 2 (14.3) | 1 (33.3) | 0 (0.0) | 3 (12.0) |
| Marginal zone lymphomas | 0 (0.0) | 0 (0.0) | 0 (0.0) | 0 (0.0) | 1 (25.0) | 1 (4.0) |
| Lymphatic plasma cell lymphoma | 0 (0.0) | 0 (0.0) | 0 (0.0) | 1 (33.3) | 0 (0.0) | 1 (4.0) |

Note. ECOG, Eastern Cooperative Oncology Group
