# Supplementary material for: Phase 1 clinical trial of the PI3Kδ inhibitor YY-20394 in patients with B-cell hematological malignancies
Source: J Hematol Oncol. 2021 Aug 23;14:130. doi: 10.1186/s13045-021-01140-z (PMC8381505; doi:10.1186/s13045-021-01140-z)
Supplement: Supplementary file 4 — Additional file 4. The methods and definition of MTD, dose escalation phase and DLT as well as hematological toxicity. [file 13045_2021_1140_MOESM4_ESM.docx]

**Additional File 4. The methods and definition of MTD, dose escalation phase and DLT as well as hematological toxicity**

With the exception of the initial 20 mg dose group (only 1), each climbing dose group (40-200 mg) was then enrolled in the "3 + 3" design with 3 - 6 patients, respectively. The maximum tolerated dose (MTD) was established during the dose escalation phase, based on dose-limiting toxicity (DLT) developments, which were defined as: death; grade IV hematological toxicity with a duration of 1 week; or non-hematological toxicities ≥ grade III. The dose below the dose at which > 33% of patients exhibited DLTs was defined as the MTD. At the end of the tolerance test, the dose extension test was conducted, in which YY-20394 was administered for a period of 28 days per cycle until the disease progressed or toxicity developed beyond tolerance. Patients visited the clinic once a week during treatment cycle 1 and every 2 weeks during cycle 2 and once a month thereafter.
