# Supplementary material for: Phase 1 clinical trial of the PI3Kδ inhibitor YY-20394 in patients with B-cell hematological malignancies
Source: J Hematol Oncol. 2021 Aug 23;14:130. doi: 10.1186/s13045-021-01140-z (PMC8381505; doi:10.1186/s13045-021-01140-z)
Supplement: Supplementary file 5 — Additional file 5.Table S3: Mean pharmacokinetic parameters of patients after a single dose in each dosage group. [file 13045_2021_1140_MOESM5_ESM.docx]

**Additional File 5: Table S3****. Mean pharmacokinetic parameters of patients after a single dose in each dosage group**

|  | **20 mg**  **(n = 1)** | **40 mg**  **(n = 3)** | **80 mg**  **(n = 14)** | **140 mg**  **(n = 3)** | **200 mg**  **(n = 4)** |
| --- | --- | --- | --- | --- | --- |
| **T_1/2_ (h)** |  |  |  |  |  |
| N (Missing) | 1 (0) | 3 (0) | 11 (0) | 3 (0) | 3 (1) |
| Mean ± SD | 27.8 | 17.8 ± 1.4 | 17.2 ± 1.8 | 18.0 ± 3.6 | 16.0 ± 1.9 |
| RSD% | - | 7.77 | 10.3 | 20.2 | 12.0 |
| **T_max_ (h)** |  |  |  |  |  |
| N (Missing) | 1 (0) | 3 (0) | 11 (0) | 3 (0) | 4 (0) |
| Mean ± SD | 4.0 | 1.8 ± 0.3 | 2.7 ± 1.5 | 2.0 ± 1.7 | 9.4 ± 10.1 |
| RSD% | - | 15.8 | 56.4 | 86.6 | 107.9 |
| **C_max_ (ng/mL)** | |  |  |  |  |
| N (Missing) | 1 (0) | 3 (0) | 11 (0) | 3 (0) | 4 (0) |
| Mean ± SD | 39.3 | 147.5 ± 24.5 | 346.8 ± 90.2 | 854.4 ± 288.9 | 640.8 ± 377.7 |
| RSD% | - | 16.6 | 26.0 | 33.8 | 59.0 |
| **AUC_0→t_ (h*ng/mL)** | |  |  |  |  |
| N (Missing) | 1 (0) | 3 (0) | 11 (0) | 3 (0) | 4 (0) |
| Mean ± SD | 1,141.7 | 2,272.8 ± 677.8 | 6,514.9 ± 1,507.2 | 14,189.7 ± 632.7 | 13,104.9 ± 3,333.7 |
| RSD% | - | 29.8 | 23.1 | 4.5 | 25.4 |
| **AUC_0→∞_ (h*ng/mL)** | |  |  |  |  |
| N (Missing) | 1 (0) | 3 (0) | 11 (0) | 3 (0) | 3 (1) |
| Mean ± SD | 1,393.0 | 2,406.5 ± 733.6 | 6,881.3 ± 1,624.4 | 15,306.0 ± 936.2 | 15,019.6 ± 2,391.6 |
| RSD% | - | 30.5 | 23.6 | 6.1 | 15.9 |
| **Vd/F (L)** |  |  |  |  |  |
| N (Missing) | 1 (0) | 3 (0) | 11 (0) | 3 (0) | 3 (1) |
| Mean ± SD | 575.5 | 445.3 ± 100.0 | 301.7 ± 70.4 | 236.0 ± 37.6 | 318.5 ± 92.2 |
| RSD% | - | 22.5 | 23.3 | 15.9 | 29.0 |
| **CL/F (L/h)** |  |  |  |  |  |
| N (Missing) | 1 (0) | 3 (0) | 11 (0) | 3 (0) | 3 (1) |
| Mean ± SD | 14.4 | 17.6 ± 4.8 | 12.2 ± 2.6 | 9.2 ± 0.5 | 13.6 ± 2.4 |
| RSD% | - | 27.0 | 21.3 | 5.9 | 17.3 |
| **MRT_0→t_ (h)** |  |  |  |  |  |
| N (Missing) | 1 (0) | 3 (0) | 11 (0) | 3 (0) | 4 (0) |
| Mean ± SD | 25.0 | 18.0 ± 0.9 | 18.6 ± 1.9 | 19.1 ± 3.6 | 21.5 ± 6.4 |
| RSD% | - | 5.1 | 10.1 | 18.9 | 29.7 |
| **MRT_0→∞_ (h)** |  |  |  |  |  |
| N (Missing) | 1 (0) | 3 (0) | 11 (0) | 3 (0) | 3 (1) |
| Mean ± SD | 40.7 | 22.4 ± 1.3 | 22.7 ± 3.1 | 24.8 ± 6.5 | 21.8 ± 4.0 |
| RSD% | - | 5.8 | 13.5 | 26.0 | 18.2 |
