# Supplementary material for: Phase 1 clinical trial of the PI3Kδ inhibitor YY-20394 in patients with B-cell hematological malignancies
Source: J Hematol Oncol. 2021 Aug 23;14:130. doi: 10.1186/s13045-021-01140-z (PMC8381505; doi:10.1186/s13045-021-01140-z)
Supplement: Supplementary file 6 — Additional file 6.Table S4: Mean pharmacokinetic parameters of patients after multiple administrations in each dosage group. [file 13045_2021_1140_MOESM6_ESM.docx]

**Additional File 6: Table S4. Mean pharmacokinetic parameters of patients after multiple administrations in each dosage group**

|  | **20 mg**  **(n = 1)** | **40 mg**  **(n = 3)** | **80 mg**  **(n = 10)** | **140 mg**  **(n = 3)** | **200 mg**  **(n = 4)** |
| --- | --- | --- | --- | --- | --- |
| **T_1/2_ (h)** |  |  |  |  |  |
| Mean ± SD | 39.5 | 19.7 ± 7.2 | 25.3 ± 25.5 | 16.4 ± 0.3 | 17.5 ± 6.3 |
| RSD% | - | 36.5 | 100.8 | 1.9 | 35.8 |
| **T_max_ (h)** |  |  |  |  |  |
| Mean ± SD | 4.00 | 2.7 ± 1.2 | 2.4 ± 1.7 | 3.7 ± 2.5 | 2.0 ± 1.5 |
| RSD% | - | 43.3 | 70.7 | 68.6 | 73.6 |
| **C_max_ (ng/mL)** | |  |  |  |  |
| Mean ± SD | 94.6 | 207.8 ± 58.3 | 529.0 ± 170.5 | 1,185.1 ± 413.3 | 1,612.1 ± 615.9 |
| RSD% | - | 28.1 | 32.2 | 34.9 | 38.2 |
| **C_min_ (ng/mL)** |  |  |  |  |  |
| Mean ± SD | 28.7 | 73.0 ± 32.5 | 180.5 ± 74.5 | 359.6 ± 31.5 | 472.4 ± 71.6 |
| RSD% | - | 44.5 | 41.3 | 8.8 | 15.16 |
| **C_ss_ (ng/mL)** |  |  |  |  |  |
| Mean ± SD | 51.0 | 132.7 ± 43.0 | 325.7 ± 97.8 | 642.3 ± 40.2 | 918.6 ± 201.7 |
| RSD% | - | 32.4 | 30.0 | 6.3 | 22.0 |
| **AUC_0→t_ (h*ng/mL)** | |  |  |  |  |
| Mean ± SD | 1,223.7 | 3,185.3 ± 1,032.5 | 7,817.8 ± 2,346.8 | 15,415.2 ± 963.9 | 22,047.2 ± 4,840.5 |
| RSD% | - | 32.4 | 30.0 | 6.3 | 22.0 |
| **AUC_0→∞_ (h*ng/mL)** | |  |  |  |  |
| Mean ± SD | 4,007.3 | 5,497.8 ± 1,627.1 | 16,608.5 ± 12,261.1 | 24,279.6 ± 1,710.0 | 36,342.8 ± 12,135.3 |
| RSD% | - | 29.6 | 73.8 | 7.0 | 33.4 |
| **Vd/F (L)** |  |  |  |  |  |
| Mean ± SD | 930.4 | 392.2 ± 227.5 | 388.0 ± 391.8 | 215.0 ± 10.0 | 239.4 ± 110.2 |
| RSD% | - | 58.0 | 101.0 | 4.7 | 46.0 |
| **CL/F (L/h)** |  |  |  |  |  |
| Mean ± SD | 16.3 | 13.4 ±3.7 | 11.1 ± 3.1 | 9.1 ± 0.6 | 9.4 ± 2.2 |
| RSD% | - | 28.0 | 28.3 | 6.2 | 23.3 |
| **MRT_0→t_ (h)** |  |  |  |  |  |
| Mean ± SD | 65.7 | 28.1 ± 10.5 | 36.4 ± 37.6 | 23.4 ± 1.5 | 25.3 ± 8.8 |
| RSD% | - | 37.4 | 103.2 | 6.3 | 34.7 |
